# Theoretical effect of hyperventilation on speed of recovery and risk of rehypnotization following recovery - a GasMan^®^ simulation

**DOI:** 10.1186/1471-2253-12-22

**Published:** 2012-09-18

**Authors:** Andre M De Wolf, Tom C Van Zundert, Sofie De Cooman, Jan F Hendrickx

**Affiliations:** 1 Department of Anesthesiology, Feinberg School of Medicine, Northwestern University Medical School, 251 East Huron, Chicago, IL, 60611-3053, USA; 2 Department of Anesthesiology, Sint-Jan Hospital, Kruidtuinlaan 32, Brussel, 1000, Belgium; 3 Department of Anesthesiology, OLV Hospital, Moorselbaan 164, Aalst, 9300, Belgium; 4 Department of Anesthesiology, OLV Hospital, Moorselbaan 164, Aalst, 9300, Belgium; 5 current affiliation: University of Maastricht, Maastricht, The Netherlands

**Keywords:** Inhaled anesthetics, Rehypnotization, Isocapnic hyperventilation, Pharmacokinetic modelling

## Abstract

**Background:**

Hyperventilation may be used to hasten recovery from general anesthesia with potent inhaled anesthetics. However, its effect may be less pronounced with the newer, less soluble agents, and it may result in rehypnotization if subsequent hypoventilation occurs because more residual anesthetic will be available in the body for redistribution to the central nervous system. We used GasMan^®^ simulations to examine these issues.

**Methods:**

One MAC of isoflurane, sevoflurane, or desflurane was administered to a fictitious 70 kg patient for 8 h with normoventilation (alveolar minute ventilation [V_A_] 5 L.min^-1^), resulting in full saturation of the vessel rich group (VRG) and >95% saturation of the muscle group. After 8 h, agent administration was stopped, and fresh gas flow was increased to 10 L.min^-1^ to avoid rebreathing. At that same time, we continued with one simulation where normoventilation was maintained, while in a second simulation hyperventilation was instituted (10 L.min^-1^). We determined the time needed for the partial pressure in the VRG (F_VRG_; representing the central nervous system) to reach 0.3 MAC (MACawake). After reaching MACawake in the VRG, several degrees of hypoventilation were instituted (V_A_ of 2.5, 1.5, 1, and 0.5 L.min^-1^) to determine whether F_VRG_ would increase above 0.3 MAC(= rehypnotization).

**Results:**

Time to reach 0.3 MAC in the VRG with normoventilation was 14 min 42 s with isoflurane, 9 min 12 s with sevoflurane, and 6 min 12 s with desflurane. Hyperventilation reduced these recovery times by 30, 18, and 13% for isoflurane, sevoflurane, and desflurane, respectively. Rehypnotization was observed with V_A_ of 0.5 L.min^-1^ with desflurane, 0.5 and 1 L.min^-1^ with sevoflurane, and 0.5, 1, 1.5, and 2.5 L.min^-1^ with isoflurane. Only with isoflurane did initial hyperventilation slightly increase the risk of rehypnotization.

**Conclusions:**

These GasMan^®^ simulations confirm that the use of hyperventilation to hasten recovery is marginally beneficial with the newer, less soluble agents. In addition, subsequent hypoventilation results in rehypnotization only with more soluble agents, unless hypoventilation is severe. Also, initial hyperventilation does not increase the risk of rehypnotization with less soluble agents when subsequent hypoventilation occurs. Well-controlled clinical studies are required to validate these simulations.

## Background

There has been a resurgent interest in the use of hyperventilation to hasten recovery from general anesthesia [1-5]. Hyperventilation during emergence allows faster removal of the potent inhaled anesthetic from the lungs and body and results in a lower alveolar concentration, but the ensuing hypocarbia decreases removal of the agent from the central nervous system (CNS) through its effect on cerebral blood flow (Q_CNS_) [4]. In order to maintain normocarbia (and thus Q_CNS_) during hyperventilation (= normocarbic hyperventilation), several techniques have been used such as the addition of exogenous CO_2_ to the fresh gases, or rebreathing of CO_2_ by reducing fresh gas flow (FGF) while the anesthetic agent is eliminated via an activated charcoal filter. But even regardless of the potential technical hurdles to maintain normocarbia during hyperventilation, is normocarbic hyperventilation even worth it in terms of speeding up recovery? We argue that the benefit of hyperventilation on speed of recovery is predictably small with modern potent inhaled anesthetics because of their low solubility [6,7]. Also, the effect of hyperventilation is likely to depend on the duration of administration of the agent [6,7]. In particular, the situation in which the muscle group (MG) would have been saturated intrigued us because a short period of hyperventilation may result in less wash-out of the agent from the saturated muscle group (MG); subsequently, when normoventilation returns or when hypoventilation occurs, the higher partial pressure in the MG could theoretically result in redistribution of a larger amount of agent to the vessel rich group (VRG; including the CNS), resulting in rehypnotization (defined as an increase in F_VRG_ >0.3 MAC). This potential detrimental effect of initial hyperventilation has not been addressed previously, theoretically nor clinically. We therefore used GasMan^®^ simulations to help demonstrate and clarify these two issues: does normocarbic hyperventilation shorten emergence substantially with modern low-soluble agents, and what is the risk of rehypnotization when subsequent hypoventilation occurs?

## Methods

GasMan^®^ (Version 4.0.0, Med Man Simulations, Inc., Chestnut Hill, MA), a computer program, is a physiologically-based model of inhaled anesthetic uptake and distribution. Agent solubility in blood and tissues, gas and blood flows, and compartment volumes (lungs, VRG, MG, and fat group) determine the rate of agent transfer [8]. The version used in this study does not correct for inter-tissue diffusion, anesthetic metabolism, dead space, or ventilation/perfusion abnormalities. In addition, several assumptions are made within GasMan^®^: increasing alveolar minute ventilation (V_A_) does not change the arterial partial pressure of CO_2_ (=normocarbic hyperventilation); F_VRG_ is representative for the partial pressure in the central nervous system, F_CNS_; the brain is a homogeneous tissue, even though gray matter and white matter have different perfusion and agent solubility; and the effect of age and other factors on MAC are ignored. MAC values that we used in our GasMan^®^ simulations are: desflurane 6%, sevoflurane 2%, isoflurane 1.2%. The GasMan^®^ program allows the user to manipulate the vaporizer setting (F_D_) of an agent of choice, FGF, circuit volume, V_A_ and cardiac output, and to observe the resulting course of inspired and alveolar partial pressure (F_I_ and F_A_, respectively) and the partial pressure of the potent inhaled anesthetic in several tissues (arterial and venous blood, VRG, MG and fat group [F_a_, F_v_, F_VRG_, F_MG_, and F_fat_, respectively]). GasMan^®^ simulations have been used to study the effect of solubility, cardiac output, and duration of anesthesia on the kinetics of the potent inhaled anesthetics during emergence [9-11].

The following GasMan^®^ program settings were kept constant for all simulations (unless stated differently): a 70 kg patient, an anesthesia breathing circuit volume of 8 L, a FGF of 1 L.min^-1^, an alveolar minute ventilation (V_A_) of 5 L.min^-1^, a functional residual capacity (FRC) of 2.5 L, and a cardiac output (Q) of 5 L.min^-1^. Other GasMan^®^ settings and parameters are presented in Table 1.

**Table 1:** **GasMan**^**® **^**settings and parameters**

	**Blood**	**VRG**	**Muscle**	**Fat**
*λ* desflurane	0.42	0.54	0.97	13
*λ* sevoflurane	0.65	1.1	2.4	34
*λ* isoflurane	1.3	2.1	4.5	70
Volume (L)		6	33	14.5
Blood flow (%)		75.8	18	6

*λ*: tissue/gas partition coefficient; VRG: vessel rich group.

In GasMan^®,^ isoflurane, sevoflurane, or desflurane were administered to attain an F_A_ of 1 MAC for 8 h, resulting in complete saturation of the VRG and near-complete saturation of the MG. Even with the low FGF of 1 L.min^-1^, F_A_ was reached fairly rapidly by using overpressure (F_D_ desflurane of 18%; F_D_ sevoflurane of 5%; F_D_ isoflurane of 3%). At the end of 8 h, with the MG >95% saturated, administration of the agent was terminated, and FGF was increased to 10 L.min^-1^ to avoid rebreathing. For each agent, we had one simulation with normoventilation (V_A_ 5 L.min^-1^) and another one with hyperventilation (V_A_ 10 L.min^-1^). We determined the time for the F_VRG_ (representing F_CNS_) to reach 0.3 MAC, which was considered to be MACawake. After F_VRG_ reached MACawake, besides continuing normoventilation and hyperventilation, several degrees of hypoventilation were instituted (V_A_ of 2.5, 1.5, 1, and 0.5 L.min^-1^), and the course of F_VRG_ was observed. An increase in F_VRG_ >0.3 MAC was considered to be evidence of rehypnotization.

## Results

**Figure 1:**
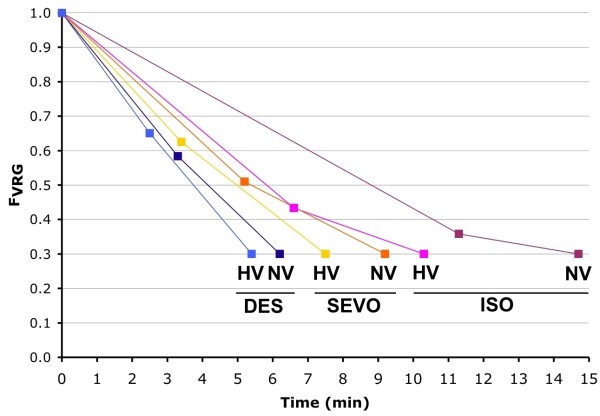
**F**_**VRG **_**during emergence.** F_VRG_ (partial pressure in the vessel rich group, representing the central nervous system) of desflurane (DES), sevoflurane (SEVO) and isoflurane (ISO) versus time during emergence with normoventilation (NV) and hyperventilation (HV). The first data point is the value of F_VRG_ at the moment F_A_ (partial pressure in the alveoli) reaches 0.3 MAC (MACawake).

Time for F_VRG_ to reach 0.3 MAC with normoventilation was 14 min 42 s with isoflurane, 9 min 12 s with sevoflurane, and 6 min 12 s with desflurane. With hyperventilation these times were reduced to 10 min 18 s, 7 min 30 s, and 5 min 24 s, respectively. Hyperventilation therefore reduced recovery times by 30, 18, and 13% for isoflurane, sevoflurane, and desflurane, respectively (Figure 1).

**Figure 2:**
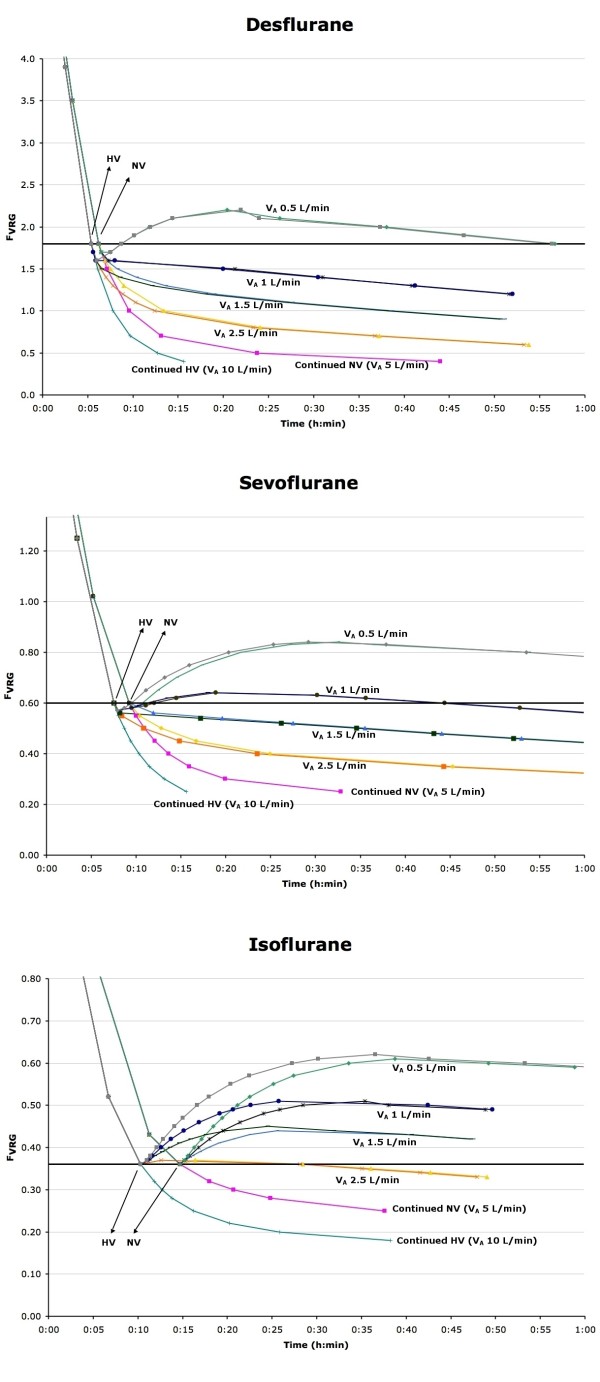
**Effect of several degrees of hypoventilation on F**_**VRG **_**during emergence.** F_VRG_ (partial pressure in the vessel rich group, representing the central nervous system) of desflurane (**Figure **2**A**), sevoflurane (**Figure **2**B**) and isoflurane (**Figure **2**C**) versus time after emergence with variable alveolar minute ventilation (V_A_). NV = normoventilation; HV = hyperventilation. Any F_VRG_ data point that is above the thick horizontal line representing MACawake indicates rehypnotization.

After normoventilation during washout, subsequent hypoventilation with desflurane slowed the further decline in F_VRG_, but only severe hypoventilation (V_A_ 0.5 L.min^-1^) resulted in rehypnotization (Figure 2A). With sevoflurane, similar observations were made except that not only V_A_ of 0.5 but also 1 L.min^-1^ resulted in rehypnotization (Figure 2B). With isoflurane, even mild hypoventilation (V_A_ 2.5 L.min^-1^) resulted in rehypnotization, and V_A_ of 1.5, 1, and 0.5 L.min^-1^ all resulted in a much faster and larger increase in F_VRG_ (Figure 2C).

In addition, initial hyperventilation before instituting hypoventilation raised F_VRG_ even higher compared to normoventilation with isoflurane (Figure 2C), but not with the less soluble agents (Figure 2A and 2B).

## Discussion

Emergence from general anesthesia with a potent inhaled anesthetic occurs in 50% of the patients when F_CNS_, reflected in the GasMan^®^ program by F_VRG_, decreases below the threshold MACawake, which we defined as 0.3 MAC. The driving force to decrease F_VRG_ is a decrease in F_a_, and GasMan^®^ assumes that at the level of the alveolocapillary interface there is virtual instantaneous equilibration between blood and alveolar gas so that F_A_ = F_a_. Therefore it is the decrease in F_A_ that results in the F_CNS_ to decrease.

Increasing V_A_ promotes the elimination of agent from the lungs, and results in a faster reduction of F_A_ and subsequently of F_a_ and F_VRG_; as a consequence, emergence will be faster. In this GasMan^®^ simulation, increasing V_A_ resulted in a small effect on emergence time with desflurane and a larger effect on emergence time with isoflurane. The effect on emergence time with sevoflurane was intermediate (Figure 1). This effect of solubility on emergence time with hyperventilation has been predicted [6,7], and can be explained by the clearance formula [12]: clearance is more affected by V_A_ (and Q) for the higher soluble agents (Table 2).

(1) $$Clearance=\frac{1}{1+\frac{\lambda_{B/G}\times Q}{V_{A}}}$$

**Table 2:** **Clearance with changing Q and V**_**A **_**(based on the clearance formula)**

**Q (L.min**^**-1**^**)**	**5**	**5**	**5**	**2.5**	**10**
V_A_ (L.min^-1^)	5	2.5	10	5	5
Clearance desflurane	0.70	0.54	0.83	0.83	0.54
clearance (% baseline)		77%	117%	117%	77%
Clearance sevoflurane	0.61	0.43	0.75	0.75	0.43
clearance (% baseline)		72%	125%	125%	72%
Clearance isoflurane	0.43	0.28	0.61	0.61	0.28
clearance (% baseline)		64%	139%	139%	64%

Q: cardiac output; V_A_: alveolar minute ventilation.

In clinical practice, hyperventilation results in hypocarbia, and this will reduce Q_CNS_. A lower Q_CNS_ will reduce the removal of the agent from the CNS, and thereby will delay emergence [4]. Therefore, unless action is taken to avoid hypocarbia, hyperventilation is not going to be very helpful in reducing emergence time. In GasMan^®^, however, hyperventilation does not result in hypocarbia and therefore Q_CNS_ is maintained, and therefore this program lends itself well to simulate isocapnic hyperventilation in patients. Our observations therefore suggest that hyperventilation techniques that include methods to avoid hypocarbia to shorten emergence time have only a marginal benefit with modern agents.

Despite the predictions that hyperventilation has little effect on emergence time with poorly soluble agents, there has been a resurgent interest in the use of hyperventilation to hasten recovery from general anesthesia, even with the less soluble agents [1-5]. These clinical studies suggest that isocarbic hyperventilation does shorten emergence. However, there may be some methodological issues with some of these clinical studies. Vesely reported a shortened emergence with isoflurane when isocapnic hyperventilation was used [1], and this is indeed what is expected: isocapnic hyperventilation reduces emergence time according to the effect of V_A_ on eliminating anesthetic from the alveoli, and this was confirmed by our simulation with GasMan^®^. However, the effect of hyperventilation may have been less impressive if the control group would not have been hypoventilated during the initial part of emergence, which could have delayed emergence [1]: the authors allowed hypoventilation to occur during the initial phase of emergence in order to increase end-expired CO_2_, thereby stimulating the return of spontaneous ventilation. Ideally the control group should have been normoventilated throughout the emergence period because even short episodes of near-apnea may result in a rapid rise of F_VRG_[13]. A second study from the same group reported a similar reduction in emergence time with isocapnic hyperventilation when sevoflurane was used [5]. This is not expected: because sevoflurane is less soluble than isoflurane, isocapnic hyperventilation is supposed to have less effect on emergence time with this agent. Once more, the authors allowed relative hypoventilation to occur in the control group. Sakata reported that concurrent hyperventilation and rebreathing to induce hypercarbia significantly shortens recovery times [2,3]. They used activated charcoal to prevent rebreathing of the potent inhaled anesthetic yet allow CO_2_ rebreathing. Surprisingly, they reported the same proportional decrease in recovery time for sevoflurane and desflurane (52% and 64%, respectively) [3]. Unfortunately, in the control group the patients were hypocarbic, which could have affected recovery time through its effects on Q_CNS_: the effect of hypocarbic vs. hypercarbic hyperventilation on recovery time has been clearly demonstrated by Gopalakrishnan: animals with hypercarbia had shorter emergence times than animals with hypocarbia [4].

Our GasMan^®^ simulation shows that hypoventilation after initial recovery not just reduces the speed of reduction in F_VRG_, but can actually increase F_VRG_ above MACawake values, resulting in rehypnotization. Lower blood and tissue solubility of the agent reduces the risk of rehypnotization when hypoventilation occurs. Eger has stated that recovery is faster with agents with lower solubility and that isocapnic hyperventilation can hasten recovery [7,10,14], and that hypoventilation can result in a rise in F_A_[7], but we believe our observations regarding the effects of subsequent hypoventilation on rehypnotization have not been described in such detail before. Rehypnotization is caused by a larger amount of agent brought into the alveoli from the peripheral tissues with venous blood than the amount of agent that is removed from the alveoli by V_A_; when F_A_ >MACawake, rehypnotization will eventually occur. The amount of agent delivered to the alveoli depends on the amount of agent released by each tissue group, and agents with higher tissue solubility may result in a larger rise in F_A_ than agents with a lower solubility. With desflurane, the low tissue solubility results in relatively less desflurane returned to the alveoli than is cleared by the lungs even in the presence of mild-moderate hypoventilation, but with isoflurane relatively more agent is released by the tissues, and therefore even mild hypoventilation results in rehypnotization.

Although initial hyperventilation could be beneficial to speed up recovery with more soluble agents (isoflurane), it unfortunately also increases the risk of rehypnotization when subsequent hypoventilation occurs, and therefore this practice cannot be recommended. Hypoventilation occurs frequently immediately after general anesthesia due to an obtunded hypoxic respiratory drive [15], excessive use of narcotics, residual muscle paralysis, or partial upper airway obstruction.

Obviously these observations based on a simulation will have to be verified by well-controlled clinical studies. In addition, the effects of hypoventilation after recovery should be studied after less lengthy procedures where the muscle group is less saturated; we would expect relatively less effect of hypoventilation after recovery on the risk of rehypnotization. Also, effects of body habitus (more or less muscle and fat mass) deserve to be studied. We can speculate that an increase in the size of the muscle group increases the risk of rehyponotization with hypoventilation after recovery, because more agent will be made available by the muscle group for redistribution.

There is a lack of published clinical data on arterial partial pressures of potent inhaled anesthetic agents during emergence. One recent study found arterial plasma partial pressure of sevoflurane 30 min after cessation of sevoflurane administration to be 20% of original arterial partial pressure (Table 2 in reference 16) - this result is consistent with the finding in our simulation (0.38/2 = 19% of original F_VRG_) at 30 min in the sevoflurane group with mild hypoventilation (Figure 2) [16].

## Conclusions

In summary, isocarbic hyperventilation to speed up emergence is theoretically less effective for the modern, less-soluble agents. It may not be worth the effort at all with desflurane. In contrast to more soluble agents such as isoflurane, when hyperventilation is used with less soluble agents such as desflurane, the risk of subsequent rehypnotization when hypoventilation occurs is virtually non-existent. Well-controlled clinical studies are required to validate these simulations.

## Competing interests

JFH has received speaker fees, including travel expenses, from the Abbott company. All of his research in the last 10 years has been funded by departmental funding only.

## Authors’ contributions

AMDW helped design the study, conduct the study, analyze the data, write the manuscript, and creation of figures. SDC helped design the study, analyze the data, and write the manuscript. TCVZ helped design the study, analyze the data, and write the manuscript. JHX helped design the study, analyze the data, and write the manuscript. All authors read and approved the final manuscript.

## Pre-publication history

The pre-publication history for this paper can be accessed here:

http://www.biomedcentral.com/1471-2253/12/22/prepub
